# Astragaloside IV Exerts Cognitive Benefits and Promotes Hippocampal Neurogenesis in Stroke Mice by Downregulating Interleukin-17 Expression *via* Wnt Pathway

**DOI:** 10.3389/fphar.2020.00421

**Published:** 2020-04-03

**Authors:** Li Sun, Heming Zhang, Wen Wang, Zhiyang Chen, Shuang Wang, Jiangjing Li, Guangyao Li, Changjun Gao, Xude Sun

**Affiliations:** ^1^Department of Anesthesiology, The Second Affiliated Hospital of Air Force Medical University, Xi’an, China; ^2^School of Basic Medicine, Air Force Medical University, Xi’an, China

**Keywords:** astragaloside IV, hippocampus, IL-17, neural stem cell, neurogenesis, stroke, synapse, Wnt/β-catenin

## Abstract

**Background:**

Stroke remains a leading cause of adult disability and the demand for stroke rehabilitation services is growing, and Astragaloside IV (As IV), a primary bioactive compound of Radix Astragali : *Astragalus mongholicus* Bunge (Fabaceae), may be a promising stroke therapy.

**Methods:**

To access the effect of As IV on adult mice after ischemic stroke, a photochemical ischemia model was established on C57BL/6 mice, which were intravenously administered As IV for three consecutive days later. And then the cognitive benefits and hippocampal neurogenesis were evaluated by Morris Water Maze (MWM) test, Golgi staining, and immunohistochemical staining *in vivo* and *in vitro*. Furthermore, to find out the underlying mechanism, interleukin-17 (IL-17) knockout (KO) mice were used, through RNA sequence (RNA-seq) analysis and immunohistochemistry. Then the mechanism of neurogenesis promoted by As IV was observed by western blot both *in vivo* and *in vitro*. Specifically, As IV, recombinant mouse IL-17A and IL-17F, and Wingless/integrated (Wnt)-expressing virus was administered respectively in neural stem cells (NSCs), and then their diameters and protein expression of Nestin, IL-17, and Wnt pathway relevant protein, were measured *in vitro*.

**Results:**

Administering As IV resulted in significant amelioration of stroke-induced cognitive deficits. And more hippocampal neurons with normal morphology, significant increments in the length of the apical dendrites, and the density of their spines were observed in As IV-treated mice. Furthermore, the immunohistochemistry staining of DCX/BrdU and Sox2/Nestin showed As IV could promote hippocampal neurogenesis and NSC proliferation after ischemic stroke, as well as *in vitro*. For the mechanism underlying, IL-17 expression was downregulated significantly by As IV treatment and knocking out IL-17 was associated with nervous regeneration and synapse repair according to the analysis of RNA-seq. Consistent to As IV treatment, knocking out IL-17 showed some promotion on hippocampal neurogenesis and proliferation of NSCs, with activating Wnt pathway after stoke. Finally, *in vitro*, NSCs’ diameters and protein expression of Nestin, IL-17, and Wnt pathway were regulated by either administering As IV or inhibiting IL-17.

**Conclusion:**

As IV stimulates hippocampal neurogenesis after stroke, thus potentially facilitates brain to remodel and repair by downregulating IL-17 expression *via* Wnt pathway.

## Introduction

Stroke remains a leading cause of adult disability and the demand for stroke rehabilitation services is growing (Stinear et al., 2020). Especially, ischemic stroke at young age is an increasing problem in both developing and developed countries due to rising incidence, high morbidity and mortality, and long-term psychological, physical, and social consequences (Boot et al., 2020). So further research is needed to better understand the underlying mechanisms of ischemic stroke and find more efficient treatment measures.

Astragaloside IV (As IV), a primary bioactive compound of Radix Astragali : *Astragalus mongholicus* Bunge (Fabaceae), has beneficial effect on ameliorating cognitive deficits after stroke through its anti-oxidant, anti-inflammatory, and anti-apoptosis properties (Li et al., 2019; Xue et al., 2019; Zhang Y. et al., 2019). Furthermore, it has been found that As IV could enhance adult hippocampal neurogenesis (Yang et al., 2017; Huang et al., 2018) and promote neural stem cells (NSCs) proliferation in transiently ischemic cerebral brains (Chen X. et al., 2019). So As IV could be a promising strategy in the therapeutic arsenal against ischemic stroke for its ameliorating cognitive deficits and promoting neurogenesis.

Some innate lymphocytes, like brain-infiltrating interleukin-17 (IL-17)-positive γδ T cells, are established as major initial IL-17 producers in acute stroke (Arunachalam et al., 2017). IL-17 is an important inflammatory factor and its over-expression indicates a poorer treatment effect and prognosis for ischemic stroke (He et al., 2019; Tian et al., 2019; Zhou et al., 2019), which always abolishes some neuroprotective effect (Sun et al., 2018; Ma et al., 2018; Zhao et al., 2019). IL-17 has a quite complex role in regulating adult neurogenesis. For example, IL-17 inhibits neural progenitor cells (NPCs) proliferation on one hand and promotes the maturation of already formed neuroblasts on the other hand (Tfilin and Turgeman, 2019). And it negatively regulates adult neurogenesis and proliferation in the dentate gyrus (DG) of adult hippocampus (Liu et al., 2014; Cui et al., 2019). However, in another study, IL-17A could maintain and augment survival and neuronal differentiation of NPCs in the subventricular zone (SVZ) after ischemic stroke (Lin et al., 2016). Therefore, the exact roles of IL-17 in regulating neurogenesis in the hippocampus after stroke have yet to be determined and need further study. Some results also suggest that IL-17 plays an essential role in inhibiting proliferation and differentiation *via* Wingless/integrated (Wnt) signaling (Shaw et al., 2016; Uluçkan and Wagner, 2016; Mansoori et al., 2017; Wang et al., 2017). Wnt signaling pathway exists widely in invertebrates and vertebrates and it plays an important role in early embryonic development, organ formation, tissue regeneration, and other physiological processes. Interestingly, As IV can not only activate Wnt signaling pathway (Cheng et al., 2016; Bao et al., 2019), but also downregulate IL-17 (Jin et al., 2017; Zhang et al., 2018).

Based on these research, it seems that there is an intimate connection among As IV, IL-17, and the Wnt signaling pathway involved in treating ischemic stroke and promoting neurogenesis. Thus, in our present study, it is hypothesized that As IV exerts cognitive benefits and promotes hippocampal neurogenesis in stroke mice, regulated by IL-17 expression *via* Wnt signaling pathway. The current study assesses As IV efficacy for the treatment of ischemic stroke. Photochemical ischemia model was established and As IV was administered intravenously (iv.). Then the cognitive deficits and hippocampal neurogenesis were evaluated by Morris Water Maze (MWM) test, Golgi staining, and immunohistochemistry staining. Furthermore, to find out the underlying mechanism, IL-17 knockout (KO) mice were used, with RNA sequence (RNA-seq) analysis, immunohistochemistry, and western blots *in vivo*. Besides, *in vitro*, As IV, recombinant mouse IL-17A and IL-17F, and Wnt-expressing virus was administered respectively into NSCs culture, and then their diameters and protein expression of Nestin, IL-17, and Wnt pathway relevant protein, were assessed.

## Materials and Methods

### Animals

All the animal experiments were performed strictly in accordance with the “Guide for the Care and Use of Laboratory Animals” by the National Institutes of Health and approved by the Animal Care Committee of Air Force Medical University (Certification No. IACUC-20180905). All mice were housed in a room maintained at a constant temperature and on a 12-h light/dark cycle (light from 08:00 to 20:00). Water and food were available at will. All animals were randomly allocated to the different experimental conditions used in this study. The IL-17 KO mice had a pure C57BL/6 background. The mice aged 4–8 weeks old were used in the experiments. All experiments were performed in age-matched mixed gender mice by experimenters blinded to the genotypes and groups.

### Photochemical Ischemia and Mouse Treatment

Focal cortical ischemia was induced by photothrombosis of the cortical microvessels as described previously (Yu et al., 2015; Yang et al., 2018). Rose bengal (Sigma, Cat# 330000) was injected intraperitoneally (ip.) at a concentration of 100 mg/kg (Yu et al., 2015). Then, a skull window was carefully made 0.3–2.3 mm posterior to the Bregma and 0.5–3.0 mm right of the midline without injuring the brain tissue. The brain was illuminated for 15 min by using a cold light source (Zeiss FL1500 LCD) of the appropriate intensity for 15 min after the rose bengal injection. To observe neurogenesis in the hippocampus, BrdU was injected ip. at a dose of 50 mg/kg once per day beginning on the second day after stroke and continuing for 6 days (Zhang Y. et al., 2019; Ryu et al., 2020). The mice were sacrificed at 7 days post-injury (dpi). For the As IV groups, 2 mg/kg As IV (Macklin, Cat# A800922) was injected iv. *via* the tail vein for three consecutive days beginning on the stroke day, according to previous research and medicine specification (Du et al., 2005; Zhang et al., 2006; Zhang et al., 2011; Xie et al., 2020). From the third day to the eighth day after stroke, the mice were tested with the MWM and then analyzed by Golgi staining and immunohistochemistry for the hippocampal neurogenesis was further observed.

### Primary Cell Culture and Treatments

For the culture of primary hippocampal NSCs, brains were removed from E12–E14 mouse embryos under a stereomicroscope (Brewer and Torricelli, 2007). The hippocampus was dissected and digested in 0.125% trypsin for 10 min at 37°C. NSCs were cultured in Neurobasal medium supplemented with 2% B27, 1% N2, 2% Gln, 20 ng/ml recombinant murine epidermal growth factor (EGF, Peprotech, Cat# 315-09), and 20 ng/ml recombinant murine fibroblast growth-basic factor (FGF, Peprotech, Cat# 450-33) for 7–10 days (Homayouni-Moghadam et al., 2018). To observe NSCs proliferation under different conditions, recombinant mouse IL-17A (IL-17A, Novoprotein, Shanghai, China, Cat# CX14) 1 ng/ml, recombinant mouse IL-17F (IL-17F, Novoprotein, Shanghai, China, Cat# CC11) 1 ng/ml, 100 nM As IV (Haiyan et al., 2016; Wang et al., 2020), or the 5 μl/ml PLDK CMV G&PR U6 Wnt2 virus (Wnt2, Neuron biotech, Lenti-3206-GR749) (Williams et al., 2016) were used, according to the product introduction. The morphology and growth feature of the NSCs were observed and taken pictures by inverted microscope after 3 days’ treatment, and collected for immunochemistry staining and western blots.

### MWM Tests

Mice were subjected to behavioral assessment beginning on the third day after stroke. The MWM test includes hidden-platform training (acquisition phase) and probe trials. Briefly, experiments were performed in a circular pool with a diameter of 145 cm. The temperature was maintained at 21–22°C. The maze was filled with water, and mice were trained to find the hidden platform during four training rounds per day with 20-min intervals between rounds for four consecutive days. Each round of training was designed as follows: upon reaching the platform, the mouse was allowed to stay on it for 10 s. If the mouse failed to touch the platform within 60 s, it was guided to the platform and allowed to stay there for 10 s. On the fifth day, the test day, the platform was removed, and each mouse was subjected to a 120 s trial to test its memory retention. The movement tracks were video recorded and automatically scored by Smart tracking software (ANY-maze; Stoelting, USA). The platform-crossing times and time and distance spent in the target quadrant were analyzed.

### Golgi Staining

Golgi staining was performed as previously described. Mice in both the control and As IV groups were perfused with 0.01 M phosphate-buffered saline (PBS, pH 7.4). The brain tissue was removed and immersed in a Golgi-cox solution (5% potassium chromate, 5% potassium dichromate, and 5% mercuric chloride) for 7 days for fixation and staining. Then, coronal sections (150–200 μm) were cut serially and washed in deionized water for 1 min. The sections were placed in 50% NH_4_OH for 30 min and subsequently in a fixing solution (Kodak; Rochester, NY, USA) for an additional 30 min. The sections were then incubated in 5% sodium thiosulfate for 10 min. After rinsing with distilled water, dehydration in a gradient of ethanol solutions was performed. The sections were finally mounted and observed under the bright field of the confocal microscope FV1000, images were taken by z-stack scanning with an excitation wavelength of 405 nm, and then the virtual color was converted into red.

### Immunochemistry and TUNEL Staining

Slides were blocked in 0.01 M PBS containing 0.3% Triton X-100 and 3% bovine serum albumin (BSA) for 1 h, and incubated with primary antibodies overnight at room temperature. The primary antibodies used were as follows: rat anti-BrdU (1: 200, Abcam, Cambridge, UK, Cat# ab6326), goat anti-Nestin (1: 500, Santa Cruz, Delaware, Cat# sc-21249), rabbit anti-Sox2 (1: 50, Santa Cruz, Delaware, Cat# sc-365823), guinea pig anti-doublecortin (DCX, 1: 600, Millipore, USA, Cat# ab2253), and rabbit anti-IL-17 (1: 500, Abcam, Cambridge, UK, Cat# ab79056). Corresponding secondary antibodies conjugated with Alexa Fluor 594 (donkey anti-guinea pig Cat# 706-585-148, or anti-rabbit Cat# 711-585-152, IgG, 1: 800, Jackson ImmunoResearch) and Alexa Fluor 488 (donkey anti-rat Cat# 712-545-153, or anti-goat Cat# 705-545-147, IgG, 1: 500, Jackson ImmunoResearch) were incubated with the sections for 2–4 h at room temperature protected from light. The nuclei were counterstained with Hoechst 33342 (1:3,000, Sigma, St. Louis).

For TUNEL/IL-17 double-staining, immunostaining of IL-17 was performed first, and then TUNEL staining followed by, according to the manual of DeadEND™ TUNEL system (Promega).

Adherent NSCs were cultured on cell slides and fixed with 4% paraformaldehyde (PFA) for 20 min. These slides were incubated with primary antibodies overnight at room temperature and with second antibodies for 2–4 h at room temperature protected from light as described above.

### Western Blot Analysis

Hippocampal tissue samples or NSCs collected were homogenized in RIPA lysis buffer. After SDS-PAGE and protein transfer, membranes were incubated with primary antibodies including rabbit anti-IL-17 (1: 1,000), mouse anti-Wnt2 (1: 5,000, Abcam, Cambridge, UK, Cat#66656-1-lg), mouse anti-Nestin (1: 1,000, Abcam, Cambridge, UK, Cat#66259-1-lg), rabbit anti-β-catenin (1:1000, Cell signaling, Cat#25362), rabbit anti-GSK-3β (1:1000, Cell signaling, Cat#12456), and rabbit anti-β-actin (1:3000, Cell signaling, Cat#5125) overnight at 4°C, followed by incubation with HRP-conjugated anti-rabbit or anti-mouse IgG (1: 5,000, Proteintech, USA) for 3 h at room temperature. Bands were visualized with an ECL kit (Thermo).

### Transcriptome Sequencing

mRNA in the hippocampus of WT and IL-17 KO mice was extracted with ESscience RNA-Quick Purification Kit (YiShan Biotech, Shanghai, China). Then the library construction and RNA-seq were performed at Shanghai Sinomics Corporation (Shanghai, China) with Illumina NovaSeq 6000 (Illumina, USA), followed by the computational analysis they provided. The criteria for differential genes was set up with P value <0.01 and fold change >1.5 or <0.5. Differential expression genes (DEGs) analysis for mRNA was performed using R package edge R. DEGs with |log2(FC)| value >1 and q value <0.05, considered as significantly modulated, were retained for further analysis.

### Image Collection and Statistical Analysis

All images of immunofluorescence staining were acquired with the BX51 and Olympus FV1000. Images were analyzed by ImageJ or Imaris software. At least three mice per group were used for comparisons. Cell counting and quantification were performed by an investigator who was blinded to the experimental design. Data are presented as the mean ± standard error. Statistical comparisons were made using Student’s t-test or analysis of variance (ANOVA) analysis. P values less than 0.05 were considered statistically significant.

## Results

### As IV Repairs Spatial Learning and Memory Abilities and Impaired Spines of Apical Dendrites in the Hippocampus After Stroke

**Figure 1:**
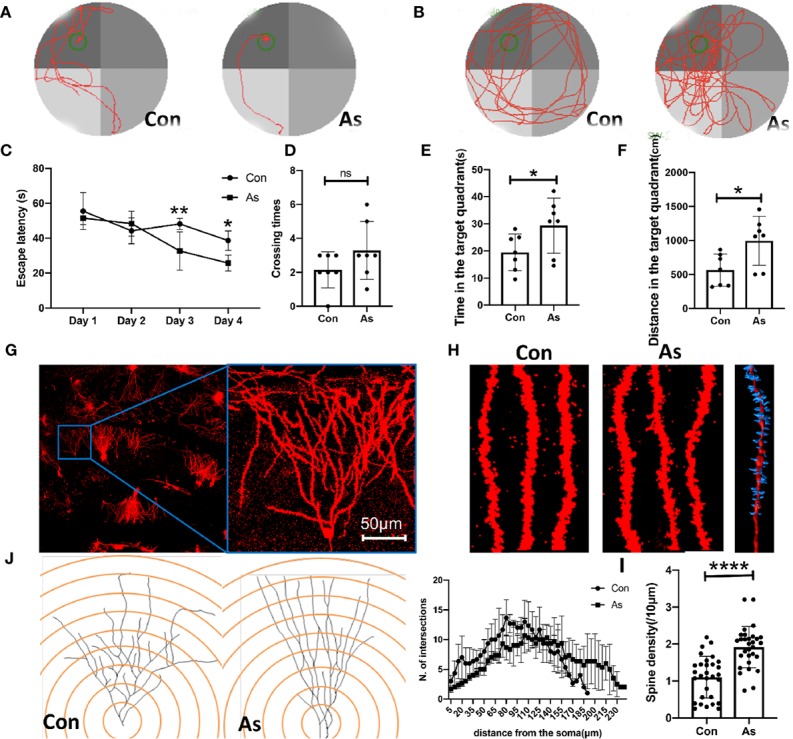
As IV improves spatial learning and memory abilities and enhances neuroplasticity in the hippocampus after stroke. **(A, B)** Representative graphs of swimming tracks on the fourth day for training and the fifth day for test in MWM. **(C)** On the third and fourth days, the mice in the As group spent less time finding the platform than those in the control group after training. n = 7. Multiple-t tests. **(D)** The numbers of crossings increased slightly on the fifth day in the mice treated with As IV. **(E, F)** On the fifth day, the mice in the As group spent more time and distance in the target quadrant. n = 7, *p < 0.05, **p < 0.01. **(G, H)** Representative dendrites and their spines in hippocampus after stroke in Golgi staining. The right graph showed the three-dimensional reconstruction for a spine in part of one dendrite created with Imaris software. **(J)** Sholl analysis was used to evaluate neuron repair in the ischemic hippocampus between the control and As groups. Notably, more neurons with normal morphology and longer distal apical branches were found in the As groups. n = 4. Multiple-t tests. **(I)** The density of spines per 10 μm in apical dendrites was increased in the As group according to Imaris analysis. n = 4, ****p < 0.0001. Con, control; As, Astragaloside IV; MWM, Morris Water Maze.

To investigate the effects of As IV on cognitive deficits and synapse repair after stroke, we injected 2 mg/kg As IV (iv.) into mice for three consecutive days. Then, all the mice were tested in the MWM assay. According to previous studies, in the MWM assay, rats treated with As IV spend less time swimming to the platform than untreated rats (Li et al., 2017; Chen P. J. et al., 2019). In this study, on the third and fourth days, the mice in the As IV group spent less time finding the platform than those in the control group (**Figures 1A, C**). On the fifth day, the mice in the As group spent more time and distance in the target quadrant (**Figures 1B, D–F**).

Few studies have focused on neuron microstructure in the hippocampus after stroke under treatment with As IV. Therefore, in the present study, Golgi staining was conducted to analyze the apical dendrites and their spines. Sholl analysis showed that more hippocampal neurons with normal morphology and longer apical branches were found in the As IV group (**Figures 1G, J**), indicating that As IV can repair the apical dendrites of hippocampal neurons after stroke. We next evaluated the density of apical dendrites’ spines. Quantification showed that the density of spines along apical dendrites was remarkably increased in the As IV group, compared with the control group (**Figures 1H, I**).

### As IV Promotes Hippocampal Neurogenesis After Stroke and Activates NSC Proliferation

**Figure 2:**
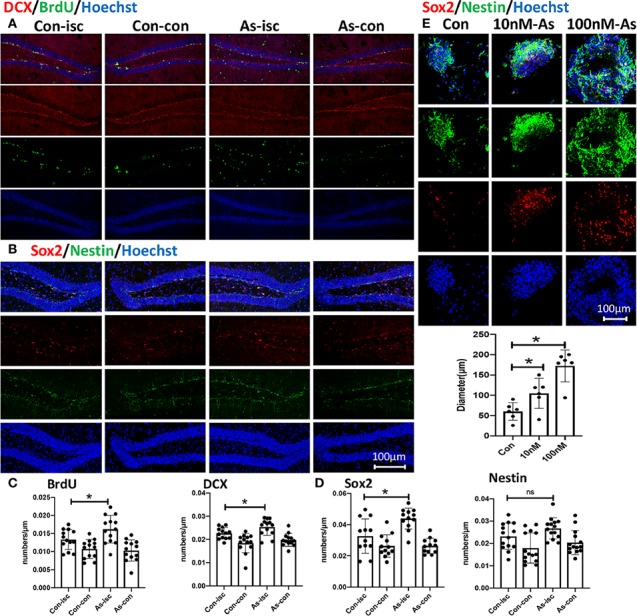
As IV promotes neurogenesis in the hippocampal DG after stroke *in vivo* and activates NSCs *in vitro*. **(A, B)** Double-immunostaining for DCX/BrdU and Sox2/Nestin in the hippocampal DG, respectively. **(C, D)** Notice significantly increased numbers of DCX-, BrdU-, and Sox2- positive cells in the As group. n = 5. *p < 0.05. **(E)** Double-immunostaining for Sox2/Nestin for NSCs. Notably enlarged diameters of NSCs were in the 10 and 100 nM As groups. *p < 0.05. DCX, doublecortin; As, Astragalosidec; Con, control; Con-isc, the ischemic hippocampus in the control group; Con-con, the contralateral hippocampus in the control group; As-isc, the ischemic hippocampus in the As group; As-con, the contralateral hippocampus in the As group; NSCs, neural stem cells; DG, dentate gyrus.

It has been shown that As IV can not only enhance adult hippocampal neurogenesis (Huang et al., 2018), but also promote NSC proliferation and neurogenesis in transiently ischemic cerebral brains after stroke (Chen X. et al., 2019). In this study, As IV promoted the expression of the DCX, BrdU, and Sox2 proteins in the subgranular zone (SGZ) after stroke *in vivo* (**Figures 2A–D**). In addition, *in vitro*, cultured primary neurospheres treated with 10 nM or 100 nM As IV proliferated better than those untreated with As IV, with sphere diameters increasing as the As IV dose increased. There were more Nestin- and Sox2-positive cells in the neurospheres composed of NSCs in both the 10- and 100-nM As IV groups than in the untreated group (**Figure 2E**).

### As IV Reverses the Overexpression of IL-17 Induced by Stroke

**Figure 3:**
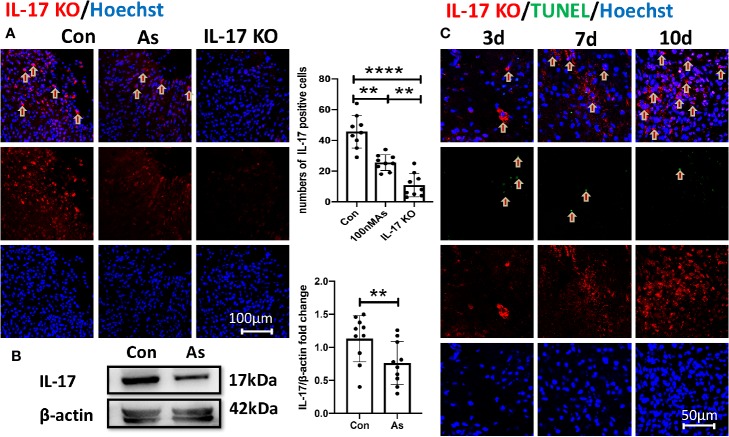
The overexpression of IL-17 after stroke is reversed by As IV. **(A)** Immunostaining for IL-17 in the infarct at 10dpi. Notice significantly decreased numbers of IL-17-positive cells of mice in the As IV and IL-17 KO groups compared with the control group. n = 3, **p < 0.01, ****p < 0.0001. **(B)** Western-blotting for IL-17 in ipsilateral hippocampus on 7dpi after stroke. Notice the significant downregulation of IL-17 by administering As IV. n = 3, **p < 0.01. **(C)** Immunohistochemistry for IL-17 and TUNEL staining in the infarct brain region of mice at different time after stroke. Notably, the expression of IL-17 increased gradually and slowly after stroke and lagged far behind the appearance of apoptosis. IL, interleukin; KO, knockout; TUNEL, TdTmediated dUTP nick end labeling; As, Astragaloside IV; Con, control.

Some results have suggested that As IV can reduce IL-4, IL-5, and IL-17 levels to inhibit asthmatic effects (Jin et al., 2017) and improve cardiac functions in children with viral myocarditis by reducing the levels of IL-17, IL-21, and caspase-3 (Zhang et al., 2018). We consistently found that the overexpression of IL-17 induced by stroke was downregulated significantly by giving As IV in both the ischemic cortex (**Figure 3A**) and ipsilateral hippocampus (**Figure 3B**). Furthermore, the expression of IL-17 increased gradually and slowly after stroke as time went on and lagged far behind the appearance of apoptosis (**Figure 3C**).

### IL-17 Is Involved in Regulating the Wnt and IL-17 Signal Pathways and Repairing Synapses in Hippocampus After Stroke

**Figure 4:**
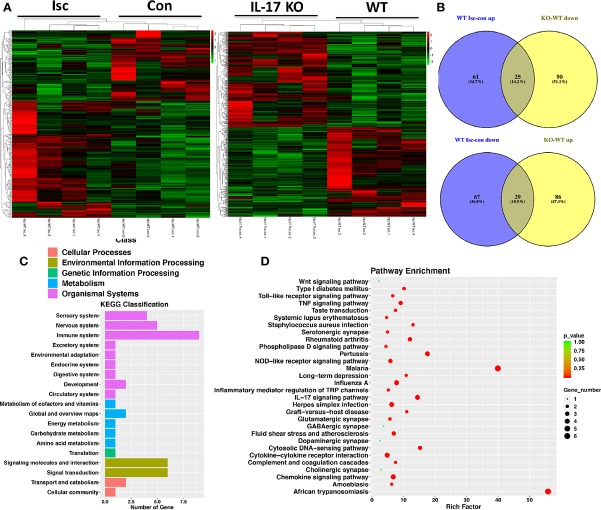
IL-17 could regulate Wnt pathway and synapses in the nervous system. **(A)** Transcriptome profiles from ischemic and contralateral hippocampus of WT mice and ischemic hippocampus from IL-17 KO and WT mice, respectively. Bands with red, black, or green in the heat map indicated high, moderate, or low expression, respectively. **(B)** The Venn diagram of DEGs by performing ischemic injury and knocking out IL-17. 25 genes were upregulated by performing ischemia but downregulated by knocking out IL-17, while 29 genes downregulated by performing ischemia but upregulated by knocking out IL-17. **(C)** The KEGG classification analysis showed DEGs in ischemic hippocampus between IL-17 KO and WT mice were closely related to the nervous and immune systems. **(D)** The pathway enrichment analysis indicated the DEGs between IL-17 KO and WT mice were involved in the regulation of Wnt and IL-17 pathways, as well as variety synapses. Isc, ischemic hippocampus; Con, contralateral hippocampus; IL, interleukin; KO, knockout; WT, wild type; DEGs, differentially expressed genes; KEGG, Kyoto Encyclopedia of Genes and Genomes.

By comparing RNA-seq from the hippocampal tissue of WT and IL-17 KO mice, differentially expressed genes (DEGs) analysis showed that IL-17 KO could reverse some genes changed by stroke, with 25 genes (Prpf8, Drd1, Arhgef25, Slc44a2, Rspry1, Nudt13, Per1, Tmem125, Fbxl19, Pik3r4, Mef2c, Mrps34, Akap13, Ccm2, Slc17a5, Grk6, Shank3, Ccdc106, Gm45736, Dhodh, Setd5, Rcbtb1, Cd52, Gtf3c1, and Extl3) upregulated by ischemia but downregulated by IL-17 KO, while 29 genes (Ip6k2, Drd1, Anks1b, Mpped1, Tpra1, Pde10a, Ryr3, Ranbp9, Mpp3, Dlg4, Fiz1, Fat1, Zeb2, Zkscan, Rik, Per1, OgtAbhd17a, Zdhhc2, Usp30, Esrrg, Gabbr1, Tmcc3, Myo6, Fbxo9, Zc3h15, Map2k7, Matr3, and Morf4l2) downregulated by ischemia but upregulated by IL-17 KO (**Figures 4A, B**). The Kyoto Encyclopedia of Genes and Genomes (KEGG) analysis showed that DEGs were relevant to the nervous and immune system (**Figure 4C**), and involved in the regulation of the Wnt and IL-17 signal pathways and the repair of serotonergic, glutamatergic, gamma aminobutyric acid (GABA)-ergic, dopaminergic, and cholinergic synapses in hippocampus after stroke (**Figure 4D**), which is consistent with the Golgi staining results in this study (**Figures 1G–J**).

### IL-17 KO Contributes to Neurogenesis and Activates Stemness of NSCs in Hippocampus After Stroke

**Figure 5:**
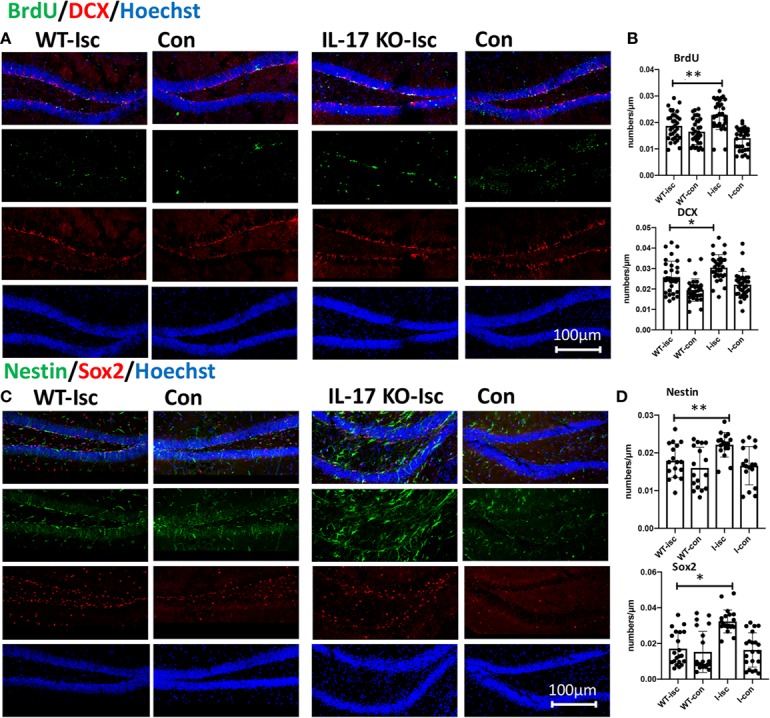
IL-17 KO contributes to promoting neurogenesis and activating stemness of hippocampal NSCs after stroke. **(A, C)** Double-immunostaining for BrdU/DCX and Nestin/Sox2 in the hippocampal DG of WT and IL-17 KO mice. **(B, D)** Notice significantly increased numbers of Nestin-, Sox2-, DCX-, and BrdU-positive cells in IL-17 KO mice compared with WT mice, respectively. n = 7, *p < 0.05, **p < 0.01. Isc, ischemic hippocampus; Con, contralateral hippocampus; WT, wild type; IL, interleukin; KO, knockout; I-isc, ischemic hippocampus of IL-17 KO mice; I-con, contralateral hippocampus of IL-17 KO mice; DCX, doublecortin; DG, dentate gyrus.

IL-17 can inhibit neuroprogenitor proliferation (Tfilin and Turgeman, 2019) and neurogenesis in the DG of the adult hippocampus (Liu et al., 2014). In this study, knocking out IL-17 significantly promoted the expression of BrdU, DCX, Nestin, and Sox2 in the SGZ after stroke *in vivo* (**Figures 5A–D**), which was consistent with the results by giving As IV above (**Figures 2A, D**).

### The Mechanism for Promoting Neurogenesis in Hippocampus After Stroke by Giving As IV and Knocking IL-17 *In Vivo*

**Figure 6:**
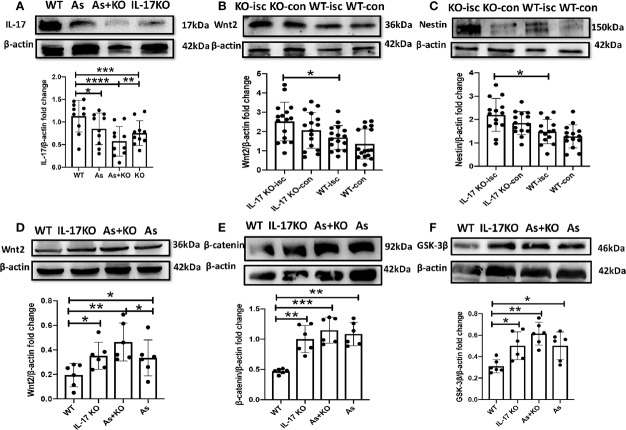
Nestin and Wnt signal pathway protein expression is upregulated by administering As IV or knocking out IL-17, and IL-17 expression is downregulated by As IV. **(A)** IL-17 expression was downregulated by giving As IV or knocking out IL-17. n = 3, *p < 0.05, **p < 0.01, ***p < 0.001, ****p < 0.0001. **(B, C)** Wnt2 and Nestin expression in the ipsilateral hippocampus was increased in IL-17 KO mice compared with WT mice at 3 dpi. n = 3, *p < 0.05. **(D–F)** Wnt signal pathway protein-Wnt2, β-catenin and GSK-3β was upregulated by treating with As IV or knocking out IL-17. n = 3, *p < 0.05, **p < 0.01, ***p < 0.001. IL, interleukin; KO, IL-17 knockout; WT, wild type; Isc, ischemic hippocampus; Con, contralateral hippocampus; As, Astragaloside; GSK-3β, Glycogen synthase kinase 3.

It has been shown that IL-17 expression is downregulated by As IV in a model of asthma (Jin et al., 2017). In this study, the protein expression of IL-17 was downregulated by either giving As IV or knocking out IL-17 (**Figure 6A**), while the protein expression of Wnt2 and Nestin in the ipsilateral hippocampus was upregulated by knocking out IL-17 after stroke *in vivo*, by western blotting (**Figures 6B, C**). And expression of Wnt signal pathway relevant protein, including Wnt2, β-catenin, and GSK-3β, was upregulated significantly by As IV treatment or knocking out IL-17 (**Figures 6D–F**).

### The NSC Proliferation Is Activated by Administering As IV or Wnt2-Expression Virus But Inhibited by Giving IL-17A or IL-17F *In Vitro*

**Figure 7:**
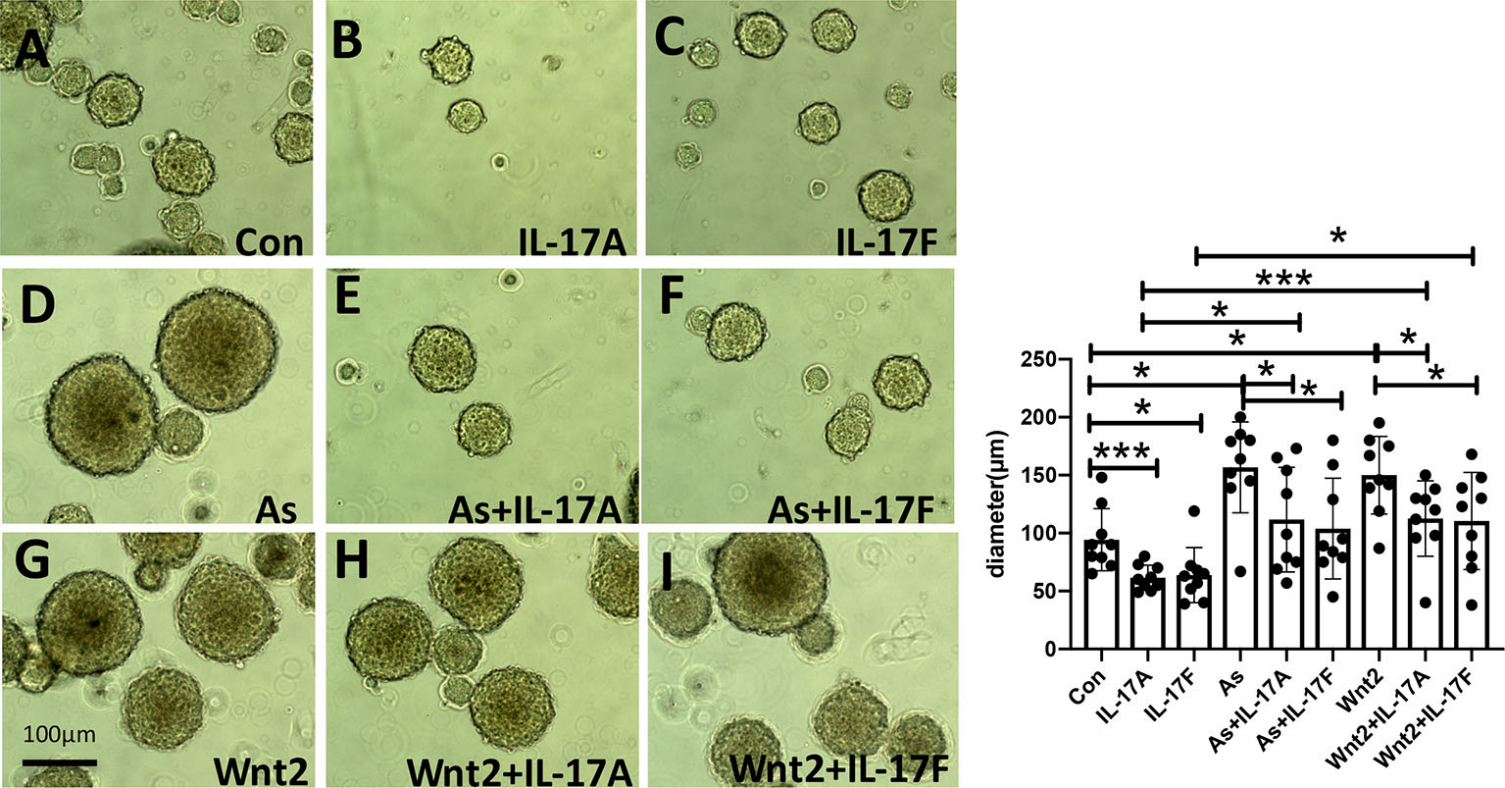
NSCs’ growth and proliferation *in vitro* is activated by administering As IV and Wnt2-expressing virus but inhibited by treatment with IL-17A and IL-17F. **(A–C, D, G)** Diameters of neurospheres were decreased by administering IL-17A and IL-17F, while increased by administering As IV or the Wnt2-expressing virus. **(B, C, E, F, H, I)** The downregulation on NSCs by IL-17A and IL-17F could be reversed by treatment with As IV and the Wnt2-expressing virus. n = 3, *p < 0.05, ***p < 0.001. Con, control; IL, interleukin; As, Astragaloside IV; NSCs, neural stem cells.

**Figure 8:**
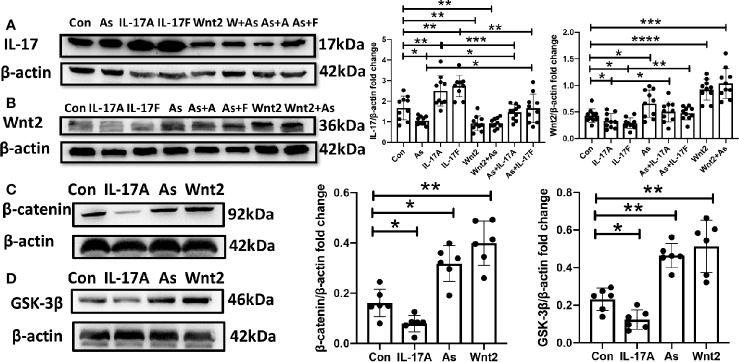
The protein expression of NSCs was regulated by As IV, Wnt2-expressing virus, IL-17A and IL-17F. **(A)** IL-17 expression was downregulated by adding As IV and Wnt2-expressing virus, while upregulated by IL-17A and IL-17F. **(B)** Wnt2 expression was upregulated by adding As IV and Wnt2-expressing virus, while downregulated by IL-17A and IL-17F. The negative regulation by IL-17A and IL-17F was reversed by giving As IV and Wnt2-expressing virus**. (C, D)** The expression of β-catenin and GSK-3β was decreased by administering IL-17A, while increased by giving As IV and Wnt2-expressing virus. n = 3, *p < 0.05, **p < 0.01, ***p < 0.001, ****p < 0.0001. Con, control; IL, interleukin; As, Astragaloside IV; NSCs, neural stem cells; GSK-3β, Glycogen synthase kinase 3.

It was said that IL-17A inhibited the osteogenic differentiation of bone mesenchymal stem cells (BMSCs) *via* the Wnt signaling pathway (Wang et al., 2017). In our study, As IV, the Wnt2-expression virus, IL-17A or IL-17F were administered to primary cultures of NSCs, respectively. By inverted microscope, we observed that IL-17A and IL-17F significantly inhibited the growth and proliferation of neurospheres (**Figures 7A–C**), while As IV and the Wnt2-expressing virus activated NSCs self-renewal (**Figures 7D, G**). Additionally, the inhibitory effects of IL-17A and IL-17F on neurospheres could be reversed by giving As IV or the Wnt2-expressing virus (**Figures 7E, F, H, I**). For western blot analysis of NSCs, IL-17 expression was downregulated by As IV and the Wnt2-expressing virus, but upregulated by IL-17A and IL-17F (**Figure 8A**). While, Wnt2 expression could significantly increase by giving As IV and the Wnt2-expressing virus and decrease when IL-17A and IL-17F was administered (**Figure 8B**). The key protein of the pathway, β-catenin, and GSK-3β, was both downregulated by giving IL-17A, and upregulated by As IV or the Wnt2-expressing virus (**Figures 8C, D**).

## Discussion

Stroke remains a leading cause of adult disability and the demand for stroke rehabilitation services is growing (Stinear et al., 2020). So, the future research of stroke must expand its horizon to find new strategies. Our study demonstrated that As IV promoted neurological function recovery and enhanced hippocampal neurogenesis after ischemic stroke. Similar to the treatment of stroke mice with As IV, knocking out IL-17 promoted the neurogenesis and neuronal plasticity in the ipsilateral hippocampus and the proliferation of NSCs *in vitro*. So, our findings indicated that As IV might become a new strategy for treating stroke and IL-17 might represent a promising biomarker for protecting the cognitive deficits caused by ischemic stroke.

As shown in present study, strategies targeting early brain damage in ischemic stroke mice by As IV can improve spatial learning and memory abilities by using MWM testing, showing that on the third and fourth days, the mice in the As IV group spent less time finding the platform than those in the control group and on the fifth day, the mice in the As group spent more time and distance in the target quadrant (**Figures 1A–F**), which is consistent with the previous study (Li et al., 2017; Chen P. J. et al., 2019). It may elicit long-term improvements in function. We further examined the morphological change about micro-structure of the hippocampal neurons. So the apical dendrites and their spines in the ipsilateral hippocampal neurons were analyzed by Golgi staining, which showed more hippocampal neurons with normal morphology, longer apical dendrites, and higher spine density along dendrites in the As IV treatment group compared with the control group (**Figures 1G–J**), indicating that As IV promotes the synaptic plasticity and repairs the impaired hippocampal neurons to exert cognitive benefits.

The mechanism underlying of neurological outcomes improvement by As IV from stroke may be due to enhanced NSCs’ proliferation and neurogenesis through activating multiple signaling pathways as reported in previous studies (Yang et al., 2017; Huang et al., 2018; Chen X. et al., 2019). As IV may facilitate the host brain neurogenesis by stimulating and activating the quiesent stem cells in hippocampus. So, immunohistochemistry staining for NPCs (Sox2 and Nestin) and neurogenesis (DCX and BrdU) were performed *in vivo* and *in vitro*. The results in this present study showed that Sox2+, DCX+, and BrdU+ cell numbers in SGZ were much higher in As IV group compared with control group *in vivo* (**Figures 2A–D**) and more Nestin+ and Sox2+ cells in the neurospheres of NSCs and enlarged diameters in both 10 nM and 100 nM As IV groups (**Figures 2E**), which indicated that As IV could significantly activate the stemness of NPCs and promote neurogenesis in the ipsilateral hippocampus after stroke.

How does As IV exert such protective effect on the ipsilateral hippocampus? We found that the expression of IL-17 in the ischemic cortex and hippocampus of stroke mice treated with As IV was decreased remarkably by both immunohistochemistry and Western blot analysis (**Figures 3A, B**) and the expression of IL-17 increased gradually and slowly after stroke as time went on and lagged far behind the appearance of apoptosis (**Figure 3C**). Consistently, previous results also suggest that the over expression of IL-17 indicates a poorer treatment effect and prognosis for ischemic stroke (He et al., 2019; Tian et al., 2019; Zhou et al., 2019), which always abolishes some neuroprotective effect (Ma et al., 2018; Sun et al., 2018; Zhao et al., 2019) and it can be reduced by As IV (Jin et al., 2017; Zhang et al., 2018). So, IL-17 expression is upregulated remarkably by ischemic stroke, which can be reversed by As IV, indicating IL-17 is a key regulation factor for As IV’s neuroprotective effect on ischemic stroke.

Moreover, for neurogenesis, IL-17 can inhibit NPC proliferation but promote the maturation of already formed neuroblasts (Tfilin and Turgeman, 2019), and the attenuation of IL-17 can promote neurogenesis in the DG of the adult hippocampus (Liu et al., 2014; Cui et al., 2019). However, IL-17A was also found to maintain and augment the survival and neuronal differentiation of NPCs in the SVZ and subsequently influence synaptogenesis and spontaneous recovery after ischemic stroke (Lin et al., 2016). Thus, the exact roles of IL-17 in regulating synapses and neurogenesis, and activating stemness of NSCs in the hippocampus have yet to be determined and need further study. Therefore, we focused on whether IL-17 played a great role in activating neurogenesis or stemness of NSCs and promoting neuroplasticity in the DG of the adult hippocampus. Similar to As IV treatment, IL-17 KO mice exhibited a remarkable stimulatory effect on the activation of NSC stemness and and enhancing neuroplasticity. For example, in our mRNA-seq analysis, 25 genes were upregulated by ischemia but downregulated by IL-17 KO, while 29 genes downregulated by ischemia but upregulated by IL-17 KO (**Figures 4A, B**), suggesting IL-17’s significant regulating effect on RNA in ischemic hippocampus. Moreover, the pathway enrichment analysis suggested that many DEGs involved in synapse plasticity were up-regulated, like serotonergic, glutamatergic, GABA-ergic, dopaminergic, and cholinergic synapses, which was consistent with the results of Golgi staining (**Figures 1G–J**), both in line with our hypothesis that As IV treatment promoted hippocampal neuronal plasticity by downregulating IL-17. Consistently, previous studies reported that Wnt signaling orchestrated expressional changes in genes encoding presynaptic and postsynaptic components in neurons (Martinez et al., 2019) and enhancing Wnt signaling could boost synaptic function during aging, and ameliorate synaptic pathology in AD (Cisternas et al., 2019; Palomer et al., 2019). Besides, in this study, DEGs between IL-17 KO and WT mice were relevant to the nervous and immune system (**Figure 4C**) and involved in the regulation of TLR, TNF, and IL-17 pathways (**Figure 4D**). Therefore, IL-17 may be a key molecule participating in neuronoplasticity in ischemic hippocampus.

Wnt signaling pathway plays important roles in early embryonic development, organ formation, tissue regeneration, and other physiological processes and As IV activates Wnt/β-catenin signaling pathway through different pathways (Cheng et al., 2016; Bao et al., 2019). Also, IL-17 could inhibit Wnt signaling (Shaw et al., 2016; Mansoori et al., 2017), or prohibit the osteogenic differentiation of BMSCs by downregulating the expression of Wnt3 and Wnt6, which can be abolished by a Wnt signaling pathway inhibitor (Wang et al., 2017). In this study, the KEGG analysis in RNA-seq showed IL-17 KO made a significant impact on Wnt pathway (**Figure 4D**). Significantly increased numbers of DCX, BrdU, Nestin, and Sox2 positive cells in SGZ were found in IL-17 KO mice, similar to As IV treated mice (**Figure 5**). For observing the regulation on Wnt signaling pathway by As IV and IL-17, western blots were conducted. The expression of Wnt2 and Nestin was upregulated significantly by knocking out IL-17 in ischemic hippocampus (**Figures 6B, C**). And the key molecular Wnt2, β-catenin, and GSK-3β expression, was upregulated by either knocking out IL-17 or administering As IV *in vivo* (**Figures 6D–F**), which was consistent with previous studies and the results of RNA-seq. Besides, in western blots, IL-17 overexpression promoted by stroke was reversed by either knocking out IL-17 or administering As IV (**Figure 6A**), consistent with the results above (**Figures 3A, B**).

*In vitro*, to observe the growth and proliferation of survival NSCs, their diameters were analyzed. We tried to observe how IL-17A, IL-17F, As IV, and Wnt2-expressing virus make effect on the diameters of NSCs. Our data showed significant enhancements in the proliferation of NSCs were induced by As IV or Wnt2-expressing virus, while remarkable decreases were observed following administering IL-17A or IL-17F (**Figure 7**). And the regulation for Wnt2 expression by giving these cytokines in western blot was similar to the change of neurospheres (**Figure 8B**). In addition, the inhibitory effects of IL-17A and IL-17F on NSCs’ diameters and Wnt2 expression could be significantly reversed by administering As IV and the Wnt2-expressing virus (**Figures 7**, **8B**). Besides, the expression of β-catenin and GSK-3β of NSCs was regulated by IL-17A, As IV, and Wnt2 expression virus (**Figures 8C, D**), whose expression change was similar to Wnt2. *In vitro*, IL-17 expression was also downregulated by adding As IV and Wnt2-expressing virus, while upregulated by IL-17A and IL-17F (**Figure 8A**).

Therefore, ample evidences, the results of RNA-seq and western blots, as well as survival NSCs’ diameters and protein expression, support that the Wnt signaling pathway is well involved in hippocampal NSC proliferation and neurogenesis, and it could be upregulated by knocking out IL-17 by giving As IV both *in vivo* and *in vitro*.

## Conclusion

In this study, As IV exerts cognitive benefits after ischemic stroke, including spatial learning and memory ability and impaired spines of apical dendrites in the hippocampus. Such cognitive protection from As IV may be due to its promotion for hippocampal neurogenesis and NSCs’ proliferation after stroke. Then the upregulation of IL-17 expression because of stroke was reduced by As IV significantly. To explore the mechanism for such neuroprotection and promoting neurogenesis, IL-17 KO mice were used to observe the RNA-seq, immunohistochemistry staining, and western blots change, showing that knocking out IL-17 or administering after stroke contributes to regulating the Wnt signal pathway, repairing variety synapses, and activating neurogenesis and NSCs’ stemness in hippocampus. Wnt signal pathway could be upregulated by both giving As IV and knocking IL-17 *in vivo*. Besides, NSC proliferation is activated by administering As IV or Wnt2-expression virus but inhibited by giving IL-17A or IL-17F *in vitro*. Thus, As IV exerts cognitive benefits and promotes hippocampal neurogenesis in stroke mice by downregulating IL-17 expression *via* Wnt signaling pathway.

## Data Availability Statement

The datasets generated for this study are publicly available, and can be found in NCBI https://www.ncbi.nlm.nih.gov/sra/PRJNA612400.

## Ethics Statement

All the animal experiments were performed strictly in accordance with the “Guide for the Care and Use of Laboratory Animals” by the National Institutes of Health and approved by the Animal Care Committee of Air Force Medical University (Certification No. IACUC-20180905).

## Author Contributions

LS, HZ, and WW were involved in designing research route, conducting related work, and writing the manuscript. ZC, SW, JL, and GL helped to collect and analyze data, and draft the manuscript. CG and XS were responsible for the idea and design of the study, and eventually approved the submitted version. All authors read and approved the final manuscript.

## Funding

This work was supported by the National Natural Science Foundation of China (Grant Nos. 81571183, 81971225, and 31570845), and by the Natural Science Foundation of Shaanxi Province (Grant Nos. 2016ZDJC-16 and 2019JQ-985).

## Conflict of Interest

The authors declare that the research was conducted in the absence of any commercial or financial relationships that could be construed as a potential conflict of interest.

The reviewer, YD, declared a shared affiliation, with no collaboration, with several of the authors, LS, WW, HZ, ZC, SW, JL, GL, CG, and XS, to the handling editor at time of review.

## Abbreviations

Wnt, Wingless/integrated; As IV, Astragaloside IV; MWM, Morris Water Maze; IL-17, interleukin-17; NSC, neural stem cell; NPC, neural progenitor cell; RNA-seq, RNA sequence; GSK-3β, glycogen synthase kinase 3; DCX, doublecortin; DG, dentate gyrus; SGZ, subgranular zone.
